# Research data management for bioimaging: the 2021 NFDI4BIOIMAGE community survey

**DOI:** 10.12688/f1000research.121714.2

**Published:** 2022-09-20

**Authors:** Christian Schmidt, Janina Hanne, Josh Moore, Christian Meesters, Elisa Ferrando-May, Stefanie Weidtkamp-Peters, members of the NFDI4BIOIMAGE initiative

**Affiliations:** 1 Enabling Technology, German Cancer Research Center, Heidelberg, Baden-Württemberg, Germany; 2 Bioimaging Center, University of Konstanz, Konstanz, Germany; 3 German BioImaging - Society for Microscopy and Image Analysis e.V., Konstanz, Germany; 4 Open Microscopy Environment Consortium, University of Dundee, Dundee, UK; 5 High Performance Computing, Johannes Gutenberg University Mainz, Mainz, Germany; 6 Center for Advanced Imaging, Heinrich Heine University Dusseldorf, Dusseldorf, Germany

**Keywords:** Research data management, bioimaging, microscopy, bioimage analysis, OMERO, FAIR-principles

## Abstract

**Background**: ****Knowing the needs of the bioimaging community with respect to research data management (RDM) is essential for identifying measures that enable adoption of the FAIR (findable, accessible, interoperable, reusable) principles for microscopy and bioimage analysis data across disciplines. As an initiative within Germany's National Research Data Infrastructure, we conducted this community survey in summer 2021 to assess the state of the art of bioimaging RDM and the community needs.

**Methods**: An online survey was conducted with a mixed question-type design. We created a questionnaire tailored to relevant topics of the bioimaging community, including specific questions on bioimaging methods and bioimage analysis, as well as more general questions on RDM principles and tools. 203 survey entries were included in the analysis covering the perspectives from various life and biomedical science disciplines and from participants at different career levels.

**Results**: The results highlight the importance and value of bioimaging RDM and data sharing. However, the practical implementation of FAIR practices is impeded by technical hurdles, lack of knowledge, and insecurity about the legal aspects of data sharing. The survey participants request metadata guidelines and annotation tools and endorse the usage of image data management platforms. At present, OMERO (Open Microscopy Environment Remote Objects) is the best known and most widely used platform. Most respondents rely on image processing and analysis, which they regard as the most time-consuming step of the bioimage data workflow. While knowledge about and implementation of electronic lab notebooks and data management plans is limited, respondents acknowledge their potential value for data handling and publication.

**Conclusion**: The bioimaging community acknowledges and endorses the value of RDM and data sharing. Still, there is a need for information, guidance, and standardization to foster the adoption of FAIR data handling. This survey may help inspiring targeted measures to close this gap.

Research highlights

• The perspectives on RDM for bioimaging were collected from participants from various scientific disciplines and at different career levels.
• Imaging core facilities play a key role in all steps of bioimaging and bioimage analysis.
• The data processing and analysis step is the most time-consuming in the bioimaging data workflow.
• Most respondents perform image analysis tasks autonomously using primarily point-and-click applications.
• Respondents acknowledge the value of reusable bioimaging data, but sharing and reuse are not widely practiced.
• Lack of knowledge, technical hurdles, and insecurity about legal aspects impede public data sharing.
• OMERO is the community's most widely used image data management system.
• Respondents endorse the value of RDM concepts, but knowledge about RDM practices and the FAIR principles is limited.

## Introduction

Imaging of biological and biomimetic specimens plays an essential role in research across many scientific disciplines. Bioimaging methods, ranging from light and electron microscopy to related photonic technologies (e.g., spectroscopy), enable the measurement and visualization of complex biological systems with high spatial and temporal resolution. They constitute key enabling technologies to test and generate scientific hypotheses. Over the past decades, the amount, size, and complexity of bioimaging data have greatly increased. ^1^ Large-scale data sets have enabled artificial intelligence- (AI) driven image analysis with highly automated workflows. ^2^^,^^3^ Significant challenges accompany these developments with respect to the storage, curation, and distribution of bioimaging data. ^4^^,^^5^ In fact, for establishing data management practices according to the FAIR (findable, accessible, interoperable, and re-usable) principles, ^6^ bioimaging as a big-data method must address similar, if not more complex, issues than those faced thus far by classical “omics” technologies (e.g., genome sequencing). Making data FAIR bears huge potential for scientific progress. A prime example of how managed, publicly shared data can have a considerable impact is the Protein Data Bank (PDB ^7^^,^^8^) which enables access to and reuse of annotated protein structure data. The most recent success story of this resource is its role in developing the AI-based prediction of protein folding from amino acid sequences. ^9^

A prerequisite for data FAIRification is the adoption of standardized or largely compatible file formats, interoperable and organized metadata, and an appropriate infrastructure for data storage and sharing. ^10^ National and international efforts to harmonize standards for research data exist both at a generic level (e.g., the Research Data Alliance RDA, GoFAIR, or FAIRsharing), and at the method-specific level. For example, the Open Microscopy Environment (OME) consortium has created solutions for bioimage data models, ^11^ data translation and data transformation (Bio-Formats ^12^), and has built the image data management system OME Remote Objects (OMERO ^13^^,^^14^). Other networks and public as well as commercial efforts have contributed data management systems, electronic lab notebooks, or tools to create data management plans. ^15^^–^^19^ Several organizations foster the international collaboration of bioimaging scientists supporting, e.g., access to high-end instrumentation ( Euro-BioImaging) and knowledge exchange ( Global BioImaging, GBI, and the network on Quality Assessment and Reproducibilty for Instruments and Images in Light Microscopy, QUAREP-LiMi ^20^^,^^21^). In Germany, the non-profit association German BioImaging - Society for Microscopy and Image Analysis e.V. ( GerBI-GMB), a network of imaging core facilities, ^22^ research laboratories, and industry partners, has recently established a joint working group on image data management together with partners from OME, called the Research Data Management for Microscopy ( RDM4mic) group. All the above-mentioned initiatives contribute to a dynamic community process that promotes bioimaging data FAIRification, including the creation of public archives (e.g., the BioImage Archive ^23^^,^^24^), added-value databases (e.g., the Image Data Resource ^25^), recommendations for bioimage metadata, ^26^ and the development of novel, cloud-ready open file formats. ^27^ Several groups worldwide have contributed tools for bioimage RDM, e.g., metadata annotation tools like the Micro-Meta-App ^28^ or MDEmic. ^29^ Web-based fora like image.sc are well established communication platforms for global exchange and facilitate the adoption of open, community-driven solutions.

Nevertheless, structured public funding programs to facilitate and coordinate the harmonization of RDM practices in the field of bioimaging are rare. They are needed to close the gap between the advancement of generic RDM concepts following the FAIR principles and the development and adoption of tailored solutions in everyday work both in biological laboratories and in imaging core facilities, where a large part of bioimage data are acquired. Since 2019, the National Research Data Infrastructure ( NFDI) is being established in Germany. The goal is to create a network of up to 30 disciplinary and method-centric consortia to “systematically manage scientific and research data” in Germany and network the data internationally ( federal state agreement, 2018). Initiated by members of GerBI-GMB, a network of research institutions and universities in Germany has formed NFDI4BIOIMAGE, a candidate consortium applying for funding within the framework of the NFDI. ^30^ This consortium aims to create and provide solutions for the management of microscopy and bioimage analysis data. To systematically assess the current status of bioimage data management and the needs of the bioimaging community in Germany and beyond, we conducted the survey presented here. The questionnaire covered various topics, from bioimaging methods and bioimage analysis to specific or generic data management tools. The results indicate that FAIR practices for bioimaging research data management are highly endorsed but not widely implemented by the bioimaging community so far. Technical hurdles, insecurity concerning legal aspects of data sharing, and a need for guidelines, training, and education are the main issues. The results from this survey constitute a resource for defining measures to address the data management needs of bioimaging scientists in a targeted manner.

## Methods

### Study design and data acquisition

We chose an analytical study design using a cross-sectional online survey with mixed question types. We drafted the questions presented in this survey inspired by the exchange between community members during the preparation phase of the NFDI4BIOIMAGE consortium and methodologically oriented on previous community surveys in the bioimaging field. ^31^^–^^34^ The questions were designed de-novo and not previously validated. Before the survey was conducted, the questions were reviewed by members of the NFDI4BIOIMAGE initiative with expertise in the relevant fields (e.g., bioimaging methods, bioimage analysis, general research data management). The questionnaire was designed with conditional logic allowing it to show a slightly different set of questions to individuals depending on previous answers. The survey logic is shown in Ext. Data 1. ^35^ A maximum of 54 and a minimum of 12 questions were asked. The survey contained yes/no, single-choice and multiple-choice questions, open field questions, and questions with preset answers on different rating scales (mostly bipolar 5-item rating scales) (Ext. Data 2 ^36^). The survey was conducted as an online questionnaire, using Machform version 11. As free, alternative software, Google Forms might be used to create a similar questionnaire, although it may not have full functionality. Participants were only allowed to participate in the survey once based on the IP address. Participants could pause and resume the questionnaire. Only completed questionnaires fully submitted by participants were included in the results, and incomplete datasets were omitted. The survey was open for participation from June, 1st, 2021 to July, 21st, 2021. (first entry: June 1st, last entry: July 19th, completed entries: 204, incomplete entries: 27; drop-out rate = 11.7 %). No target sample size was defined a priori. The full annotated questionnaire is available as Ext. Data 2. ^36^

### Participants

The survey could be accessed through the NFDI4BIOIMAGE website without restriction. No registration or sign-up was required. The survey was announced via different channels by providing the link to the survey entry page on nfdi4bioimage.de. The link was shared via the newsletter of German BioImaging – Gesellschaft für Mikroskopie und Bildanalyse e.V. (GerBI-GMB), which addresses researchers, companies, core facilities and institutes within GerBI-GMB, and their respective users. Additionally, the link to participate in the survey was shared with participants at the ELMI conference 2021, on the NFDI4BIOIMAGE website, via the Confocal Mailing List, and at the Euro-BioImaging Virtual Pub on June 4th, 2021. To mitigate a sole bias towards members of the bioimaging community closely associated with GerBI-GMB, we asked the spokespersons of established and planned NFDI consortia to distribute the invitation within their communities. These consortia cover a wide range of scientific disciplines. The survey was furthermore announced in three posts on Twitter. The survey language was English.

### Ethics and consent

No person-specific data was collected, except for the IP-address, which was temporarily stored, deleted before analysis and is not published. To participate in the online questionnaire, participants were required to provide informed consent to the data collection and anonymous processing and publication of the data on the introduction page before starting the survey (see Ext. Data 2 ^36^). Data privacy protection information was provided to the participants, including information on the legal basis, the responsible person, and the right to review and withdraw personal data. Participants were offered the option to abort their participation. Since participation in the survey posed no risk to participants’ health or personal data, no ethical approval was required per institutional guidelines.

### Data analysis

The data was analyzed in Microsoft Excel 2019. Graphs were generated in Microsoft Excel 2019. Figures were post-processed and assembled in Inkscape and Affinity Designer. The anonymized raw data is available in csv-format (Ext. Data 3 ^37^). The xlsx-file with the anonymized raw and analyzed data is available as Ext. Data 4. ^38^ Before data analysis, the survey data was subjected to quality control. Exclusion criteria were defined to omit data either partially or fully, if quality criteria were not met. We included criteria for validation of attentive participation using attention check questions (see Ext. Data 2 ^36^). For multiple-choice questions with five-item scales, the variance of answers per question was monitored per participant. If the variance was zero, i.e., all answers were equal for a given set of questions, this was interpreted as non-attentive clicking and the data set part was excluded from the analysis. The full set of exclusion criteria, explanations on each quality check, and the number of fully or partially excluded data per exclusion criterion is available in Ext. Data 1. ^35^ Omitted data is marked in the analysis xlsx-sheet as “DELETED“. Internal color-codes were used to mark entries during analysis. Review the sheet-internal notes for details. The correlation analysis described in Suppl. Figure 11 ^35^ was performed by transforming the answer items to an ordinal scale (5 = I fully agree, 1 = I disagree fully) and calculating the Spearman’s rho and p-value (using JASP version 0.16) with data from all participants who answered both questions. The free-text comments on questionnaire pages 16 and 18 were detached from their original entry number and are listed separately to ensure that respondents are not identifiable based on their comments.

*References included in the questionnaire*

The following resources were named in questions in this survey:

*(Image) data management (and analysis) systems:* OMERO, ^39^ Cytomine, openBIS, ^16^ CATMAID, ^40^ FAIRDOM-SEEK, ^17^ iRODS, BisQue ^41^

*Image Analysis tools and software:* ImageJ, Fiji, ^42^ Imaris, Huygens, Python, scikit-image, Cell Profiler, ^43^ Ilastik, ^44^ Icy, Clij/Clij2, ^45^ Napari, ^46^ KNIME, Neuralab

*Repositories:* Harvard Dataverse, Dryad, Figshare, Plant Genomics and Phenomics Research Data Repository, The Human Protein Atlas, ^47^ Allen Cell Explorer, EBRAINS, Cell Image Library, YRC Public Image Repository, Journal of Cell Biology Repository, (discontinued), EMPIAR, ^48^ Riken SSBD database), BioStudies, IDR, ^25^ BIA. ^24^

## Results and discussion

### The survey data represents the views from a wide range of scientific disciplines and career levels

**Figure 1.**
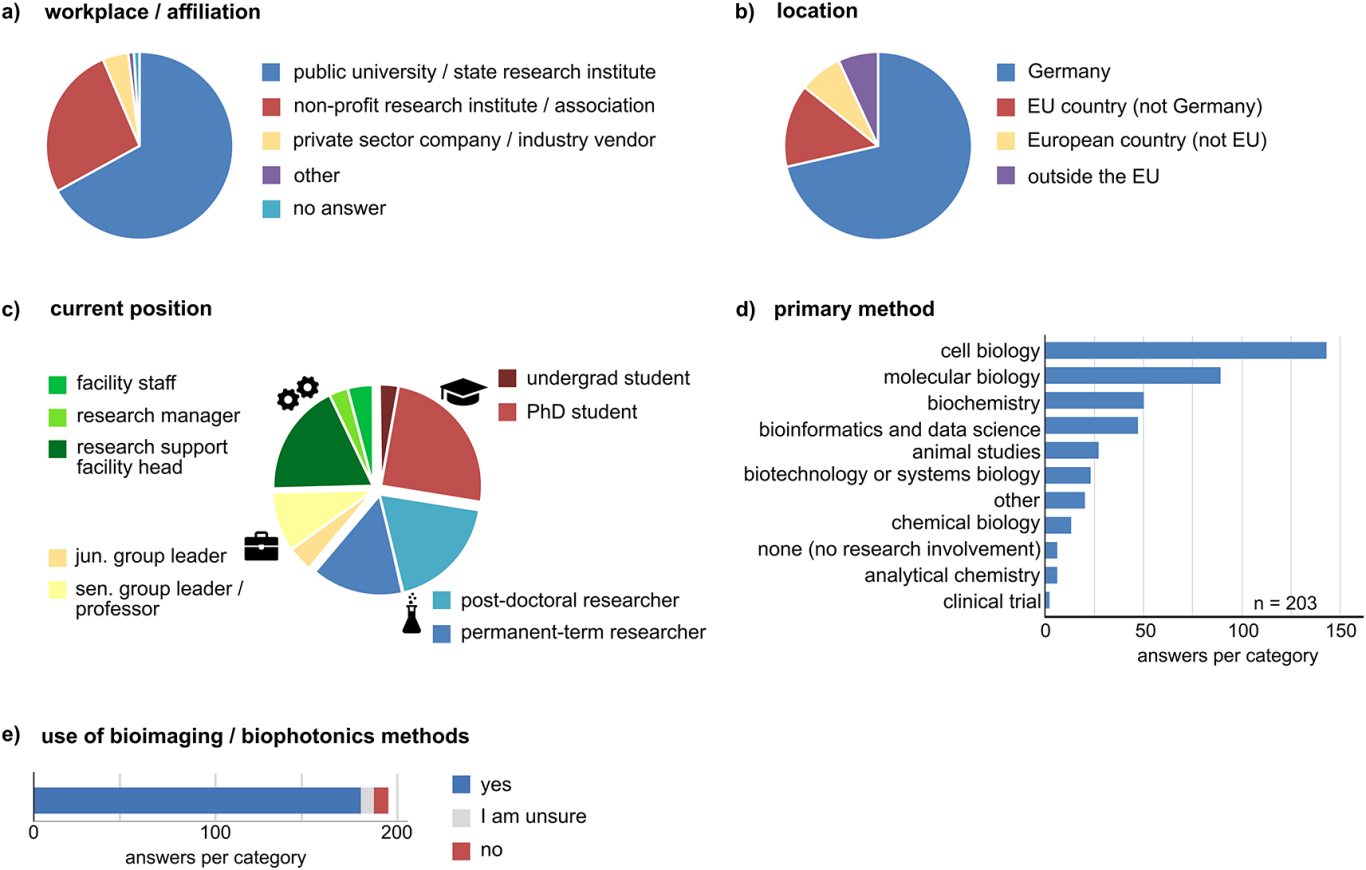
Overview of the survey respondents. Shown are responses for (a) “I work at/I am affiliated with”, (b) “My current (primary) position is located in”, and (c) “My current primary position is”. The latter criterion was used to distinguish four different groups i) undergraduate and PhD students, ii) postdoctoral and permanent-term researchers, iii) junior and senor group leaders, and iv) research support and facility staff. n = 198, five respondents are not included (two stated “Consultant” and “Company”, three left the field blank). The symbols used in c represent these groups throughout the manuscript. d) Participants were asked to state which approaches describe their work best (multiple answers possible). e) Number of participants stating to use, not to use, or to be unsure if they use bioimaging methods in their work.

In total, 204 participants completed the survey. The drop-out was low (27 incomplete surveys, 11.7%). One entry was invalid according to the exclusion criteria (Ext. Data 1 ^35^). We included 203 entries into the analysis. The majority of respondents work in Germany (145), and participants from outside Germany were distributed equally over EU and non-EU countries. Most participants work at universities or public institutes, ~27 % work at non-profit research institutes, and only a minority elsewhere ( Figure 1a, b). The entries represent a heterogeneous group with respect to the career and experience levels (primary position). We used this criterion to check if different answer patterns were observed between four distinct groups: i) undergraduate and PhD students (n = 55), ii) postdoctoral researchers and permanent research staff (n = 66), iii) junior and senior group leaders or professors (n = 27), and iv) research support and facility staff (incl. heads of facilities, research managers, n = 50) ( Figure 1c, the symbols shown in this figure are consistently used throughout the manuscript). One person of the junior/senior group leaders stated to work at a governmental institute in Germany. One person from the research support staff stated to work for a data initiative. Five entries were not included in the subgroup analysis (three left the answer field blank, two were from private sector companies). To further characterize the participants, we asked about their main fields of work and which methodological approaches would best describe their work. The participants could choose up to three items to describe their field of research (average: 1.7 items per person), which included a wide variety of areas, most frequently human biology/preclinical medicine, animal biology, biophysics, immunology, and neuroscience (Suppl. Figure 1 ^35^). A large fraction of participants chose research support as part of their research field, most prominently in the group of research support and facility staff. Plant biology is overrepresented in the group of junior and senior group leaders (Suppl. Figure 1 ^35^). Most participants use methods from cell biology, molecular biology, and biochemistry ( Figure 1d). About 90% of the respondents stated to use bioimaging or biophotonic methods in their work. A small fraction (~3.5%) declared to be unsure if they use bioimaging methods ( Figure 1e). Hence, the survey data includes the perspectives of many stakeholders in bioimaging including researchers at all career levels and research support staff.

### Confocal (fluorescence) light microscopy remains the leading bioimaging technique in use. Data processing and analysis is the most time-consuming step

**Figure 2.**
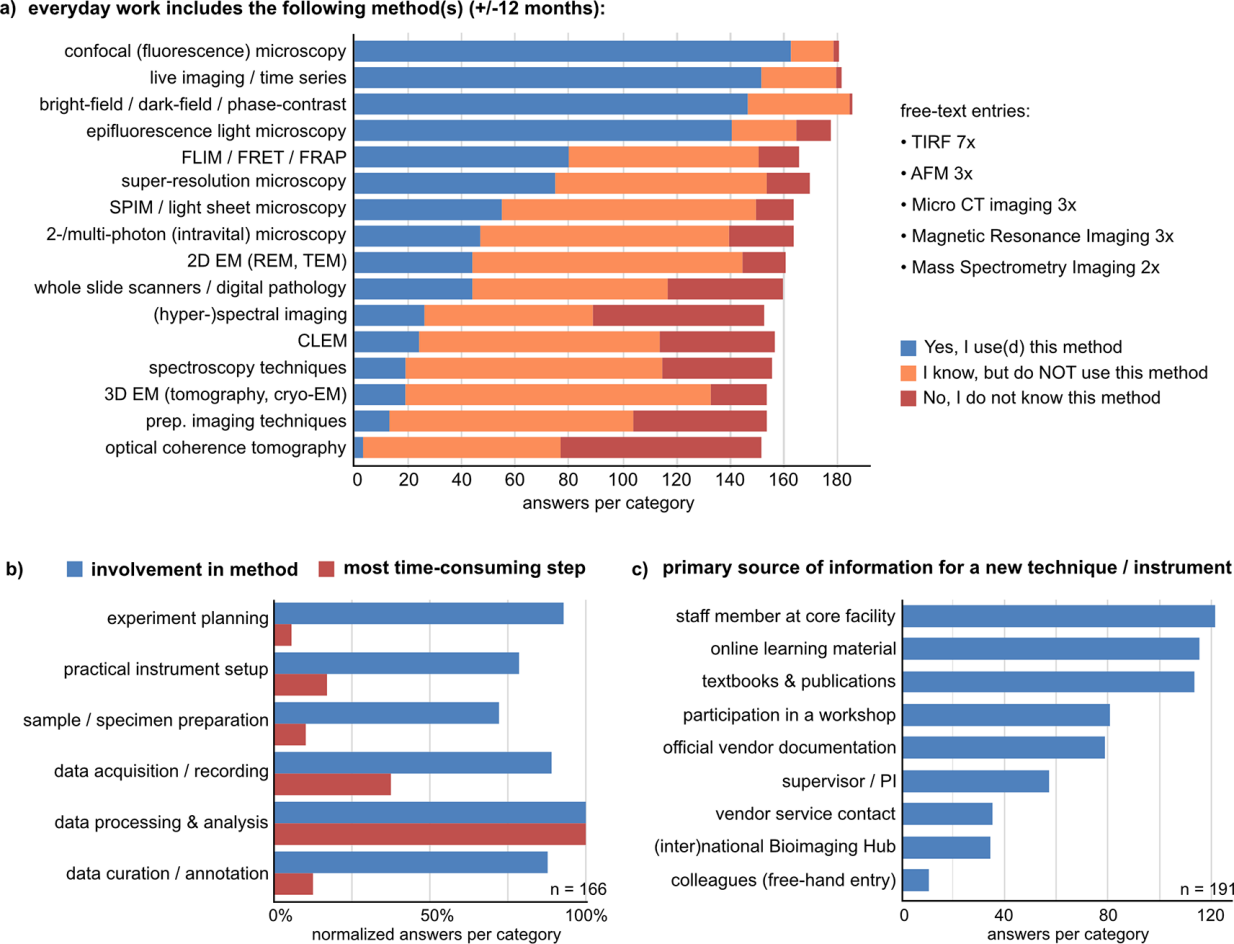
Knowledge and use of bioimaging methods. a) Participants were asked to state, if their work includes the indicated methods (± 12 months) with a preselected list and a free-text option. This question was shown to respondents who stated “I use bioimaging or biophotonics methods” or "I am not sure, if one of my methods is a bioimaging or biophotonics method”, incl. one person who left the field blank ( Figure 1e, n = 188). b) Participants could choose one method as the most important for their work (Suppl. Figure 3 ^35^), for which we asked in which aspect(s) of the method they are involved (blue bars, multiple choice) and which step is the most time-consuming (red bars, single choice). c) Participants were asked about their main information source(s) for learning a new bioimaging method (multiple choice). See also Suppl. Figure 4. ^35^ Abbreviations: AFM Atomic Force Microscopy; CLEM Correlated Light and Electron Microscopy; FLIM Fluorescence Lifetime Imaging; FRAP Fluorescence Recovery After Photobleaching; FRET Förster Resonance Energy Transfer; SPIM Selective Plane Illumination Microscopy; REM Reflection Electron Microscopy; TEM Transmission Electron Microscopy; TIRF Total Internal Reflection.

To inquire which bioimaging techniques are most widely known and most often used, we presented a list of preselected methods asking if the participants use, know, or don’t know the respective method ( Figure 2a). In the questionnaire, “using a method” was defined as being involved in at least one of six aspects: 1) experiment planning, 2) sample/specimen preparation, 3) instrument setup, 4) data acquisition and recording, 5) data processing and analysis, and 6) data curation and annotation. We asked retrospectively about work performed in the last 12 months and plans for the next 12 months. The most frequently used methods were confocal fluorescence microscopy, bright field/dark field/phase contrast microscopy, epifluorescence light microscopy, and live imaging. Advanced imaging techniques like super-resolution microscopy, fluorescence lifetime imaging (FLIM), Förster resonance energy transfer (FRET), or fluorescence recovery after photobleaching (FRAP) are used by ~40% and known by ~80% of respondents. Additional methods of importance could be added as free-text ( Figure 2a). Overall, knowledge about and use of bioimaging methods was highest in the research support group and lowest among undergraduate and PhD students (Suppl. Figure 2 ^35^).

To find out which methods would be mostly represented by the survey answers, we asked participants to choose one most important method if possible. 79 of 181 respondents chose confocal microscopy as the most important technique, mainly based on the frequency of use in their work. 23 participants stated that choosing one would be impossible (Suppl. Figure 3 ^35^). Therefore, the data are partially skewed towards well-established and widely distributed bioimaging methods like confocal imaging, which might not allow resolving specific data management needs of single, less common advanced bioimaging techniques. Higher participant numbers in follow-up surveys might allow to further distinguish method- or profession-specific needs. For the most important technique, we asked which steps respondents were involved in and which step was most time-consuming ( Figure 2b; n = 166). 83 respondents stated to be involved in all aspects of method use, and 32 were involved in all but one aspect. In contrast to this relatively uniform distribution, data processing and analysis was pinpointed as the one most time-consuming step ( Figure 2b), while the average time spent on the technique in total is mostly between 1-6 h per week and 1-6 h per day (Suppl. Figure 3 ^35^).

When participants learn a new bioimaging technique, the three most often used information sources overall were 1) staff members at the core facility, 2) online learning material, and 3) textbooks and publications ( Figure 2c). In particular, early career and permanent-term researchers most strongly rely on core facility staff (Suppl. Figure 4 ^35^). Group leaders/professors stated textbooks and publications as well as online learning material as their primary sources of information. Respondents from the research support group most frequently stated taking part in dedicated workshops and have the highest relative fraction of participants who use national or international bioimaging hubs as an information source (Suppl. Figure 4 ^35^). These results indicate that core facilities are indispensable for disseminating bioimaging know-how and are also crucial for building bridges to international resources.

### Most respondents use open source “point-and-click” software for bioimage analysis. Bioimage analysis experts prefer automated analysis pipelines and use a wider array of tools

**Figure 3.**
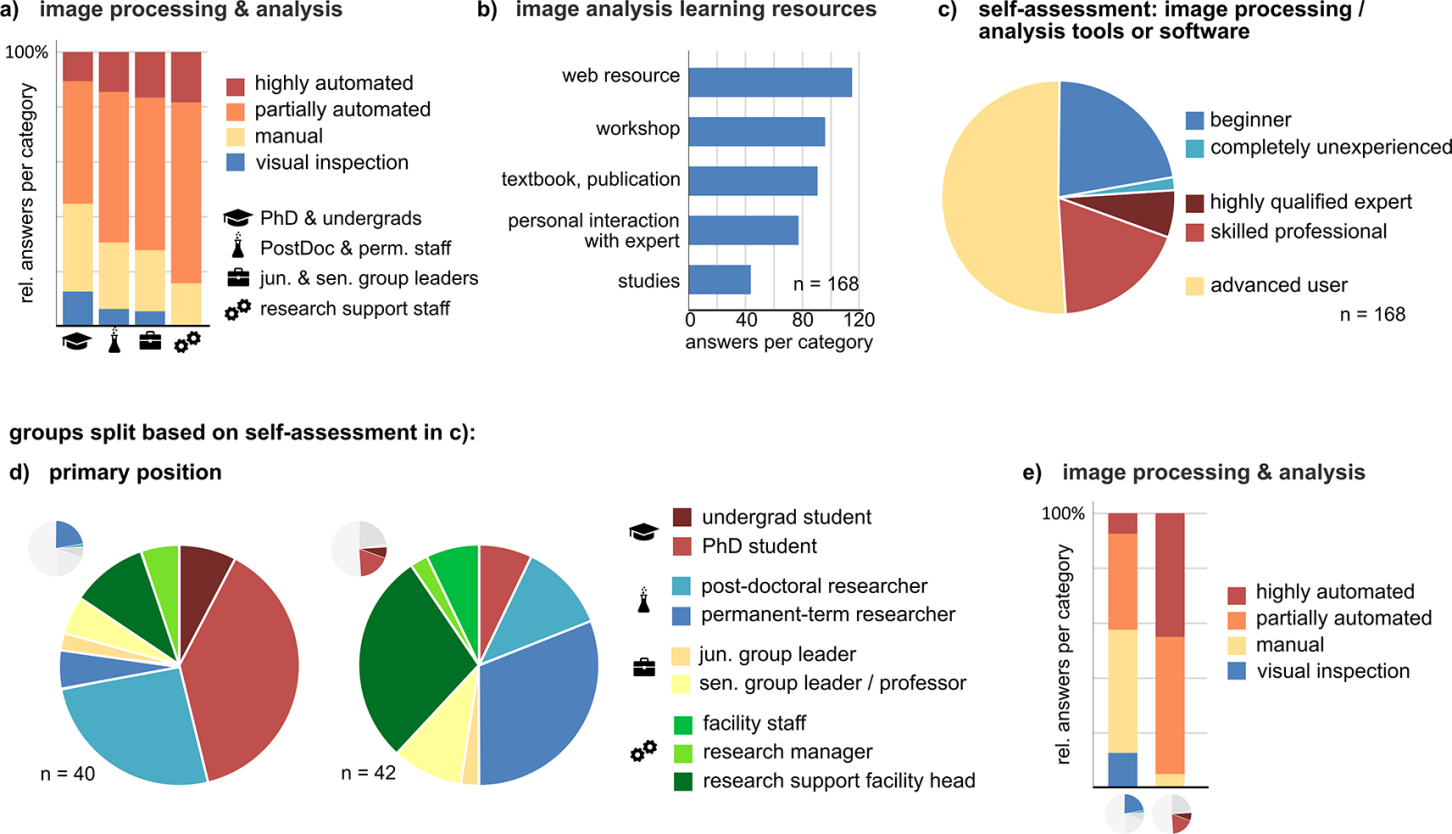
Role of bioimage analysis and aspects of autonomous processing and analysis. ^35^ a) Mode of image analysis by autonomous users and (b) their sources information for learning bioimage analysis procedures (n = 168). c) Self-reported skill levels in bioimage analysis of autonomous users, and (d, e) comparison between skilled professionals/qualified experts (n = 42) vs. inexperienced and beginners (n = 40).

Most, if not all, bioimaging data are subjected to processing and analysis. Therefore, we were interested to learn more about this aspect of the bioimage data life cycle. Of the 192 respondents on this survey page, 185 perform image analysis and processing, mostly on their own or within their research group (Suppl. Figure 5a ^35^), consistent across all groups. Depending on their answers, participants were asked either about their personal knowledge and use of image analysis (autonomous, Figure 3) or their knowledge of the collaboration partner’s use of image analysis. Autonomous performers solve bioimage analysis tasks in a heterogeneous way, ranging from manual inspection to fully automated workflows. The relative fraction of partially or fully automated image analysis as opposed to manual image analysis or visual inspection increases with career level ( Figure 3a). Self-education plays a crucial role in learning how to perform bioimage analysis ( Figure 3b). In line with the 2020 survey of the NIH Center for Open Bioimage Analysis (COBA), ^31^ the most frequently and autonomously used image analysis tools are open-source and proprietary ‘point-and-click’ applications, mostly on local computers (in particular ImageJ or Fiji; Suppl. Figure 5b, c ^35^). According to our survey, the second most often used software was IMARIS, which was, however, chosen less than half as often as Fiji/ImageJ. We used the self-reported skill level of autonomous users to compare beginners and inexperienced users with professionals and experts ( Figure 3c). Higher skill level was primarily reported by advanced career or permanent researchers and research support/facility staff. In addition, skilled autonomous users employ fully or semi-automated bioimage analysis workflows much more frequently, while beginners rely more on manual image analysis or visual inspection ( Figure 3d and e). In addition, a higher skill level correlated with a wider array of used software and knowledge about image analysis methods (Suppl. Figure 5d, e ^35^). Self-reported experts and beginners were differently distributed across research disciplines, and experts primarily stated to work in research support (Suppl. Figure 5f ^35^).

A total of 35 participants stated that an external collaboration partner or core facility specialist performed bioimage analysis on their data. Of these, 17 do not perform image analysis on their own, and 18 stated that, in addition to the analysis performed by them, a significant part of their data is analyzed by a collaboration partner (see Suppl. Figure 5a ^35^). Respondents resorted to external partners because of a lack of expertise, lack of necessary software or hardware resources, and because of established collaborations that reduced the workload (Suppl. Figure 6a ^35^). External partners use more frequently fully automated or semi-automated image analysis than participants performing the tasks on their own (Suppl. Figure 6b ^35^). About half of the respondents relying on external bioimage analysis state that the tasks require dedicated compute clusters, most often provided by the institution (Suppl. Figure 6c ^35^). Data sharing with the collaboration partner is mostly achieved via institutional cloud storage, but occasionally also via commercial cloud providers or by e-mail. Some respondents send their data by mail on a hard drive (Suppl. Figure 6d ^35^). Participants report that their external collaboration partner(s) use ImageJ/Fiji most often. While the group size is much smaller compared to participants performing image analysis on their own, relatively, the fraction of other image analysis software use is higher among external collaboration partners than in the autonomous performer group (Suppl. Figure 6e ^35^).

In sum, bioimage analysis is rated as highly important, requiring means for proper data handling and exchange, as well as documentation to ensure reproducibility of analysis steps.

### Data management systems are requested by the community but are not widely implemented. OMERO is the best known and most widely used image data management platform

**Figure 4.**
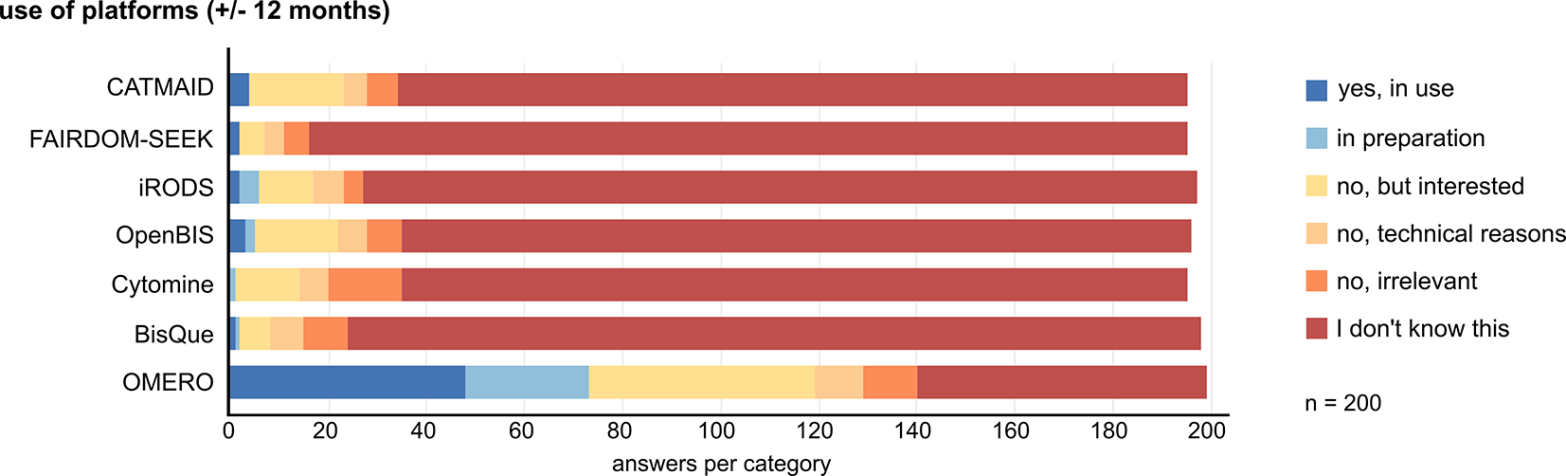
Data management platform knowledge and use by participants. We presented a list of generic and image-data-tailored management systems and asked respondents for their use, interest, and knowledge about each system on the indicated scale. Abbreviations: CATMAID Collaborative Annotation Toolkit for Massive Amounts of Image Data; FAIRDOM-SEEK Findable, Accessible, Interoperable and Reusable Data, Operating procedures and Models; iRODS Integrated Rule-Oriented Data System; OMERO Open Microscopy Environment Remote Objects; OpenBIS Open Biology Information System.

Bioimage data storage and handling after acquisition, but also the documentation of data provenance during processing and analysis, are essential aspects of FAIR data handling that require dedicated tools. We wanted to know if and which data management tools are used by the community. We preselected a set of generic and imaging-specific data management systems and asked participants whether they know, use, or plan to use any of these systems within ± 12 months. The answers revealed a clear dominance of OMERO as the best known and most frequently used platform. Only 48 of 200 respondents have OMERO in use, and another 25 are preparing to use OMERO. 46 stated to be interested in doing so. Any other data management platform was only known, used, or planned for use by less than 36 of 200 participants ( Figure 4). The presented systems come with different functionalities (e.g., federations for data sharing in iRODS), are tailored to specific fields (e.g., Cytomine for histology image analysis), and have been developed and distributed over different time periods, partially explaining the different frequencies of use. Despite the limited use, survey participants widely acknowledge the usefulness of a bioimage data management system for data organization, facilitation of publication, and increasing reproducibility. However, there is no clear tendency regarding the effort-to-benefit ratio of implementing a data management system (Suppl. Figure 7 ^35^).

**Figure 5.**
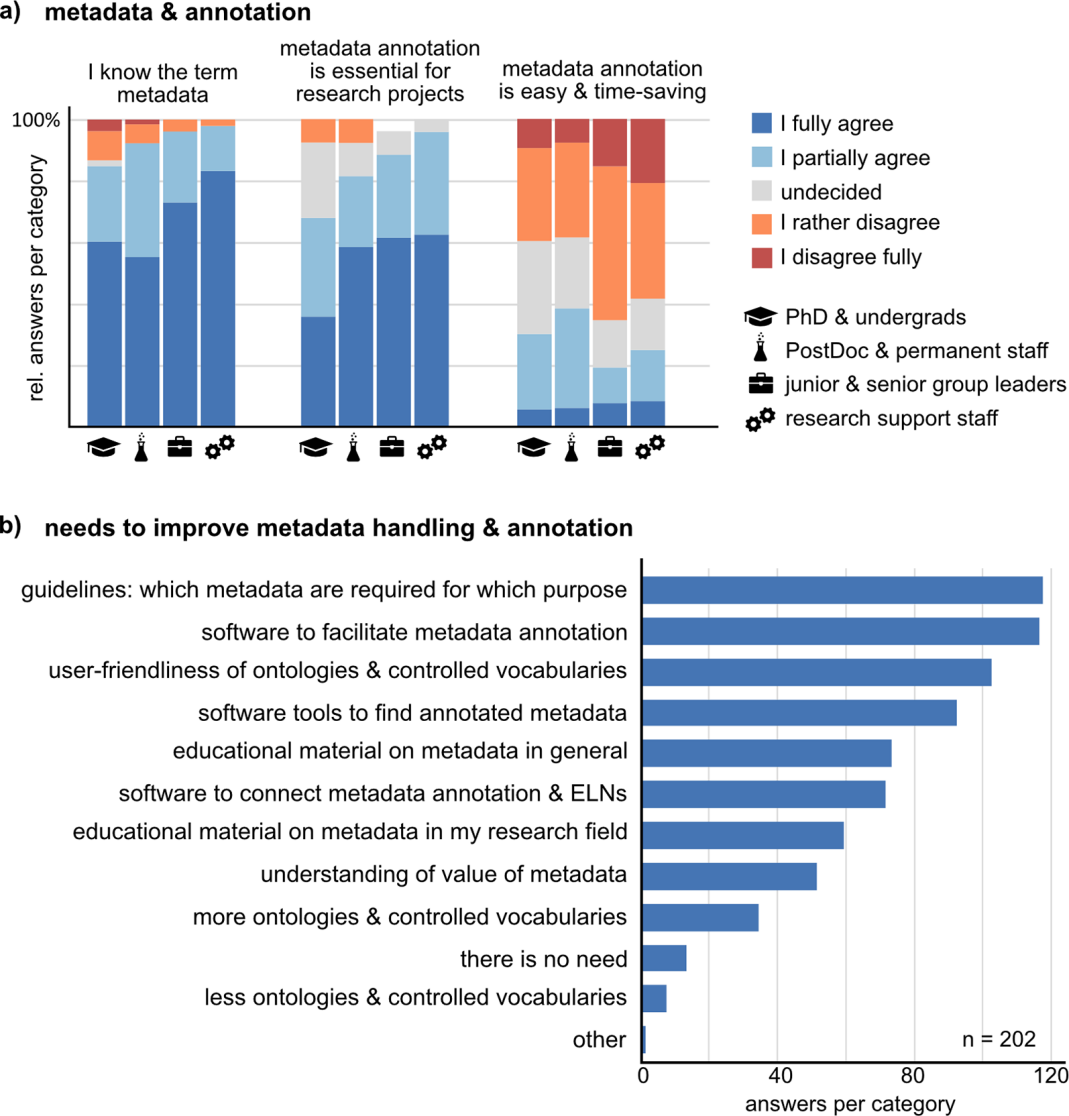
The role of metadata for research data management and the needs for metadata annotation. a) Respondents stated their opinion about three statements on metadata (“The meaning of the term “metadata” is clear to me”, “Systematic and exhaustive metadata annotation is essential for data management in a research project”, and “Systematic and exhaustive metadata annotation is easy and timesaving”) on a five-item scale. The bar graphs show the relative fraction per answer-item in each of the four groups (undergraduate and PhD students, n = 55; Postdoctoral and permanent researchers, n = 66; junior and senior group leaders/professors, n = 27; Research support staff, n = 48). b) Participants were asked to state up to three most urgent needs to improve metadata handling and annotation. Abbreviation: ELN Electronic Lab Notebook.

Data management systems – among other functions – allow users to organize data in conjunction with its metadata which is essential to preserve all necessary information about the experiment to understand and reuse the data. Moreover, metadata can allow machine readability and interoperability. We asked the respondents about their metadata handling and annotation. While respondents know the meaning and acknowledge the importance of metadata for bioimage data management ( Figure 5a), tools and guidelines are missing to make metadata annotation easier and more time-saving ( Figure 5b). Many respondents state that they do not collect metadata in addition to the automatically saved instrument metadata and if so, they use individual annotation formats with little standardization (Suppl. Figure 8 ^35^).

### Limited experience with data management plans (DMPs) and electronic lab notebooks (ELNs)

**Figure 6.**
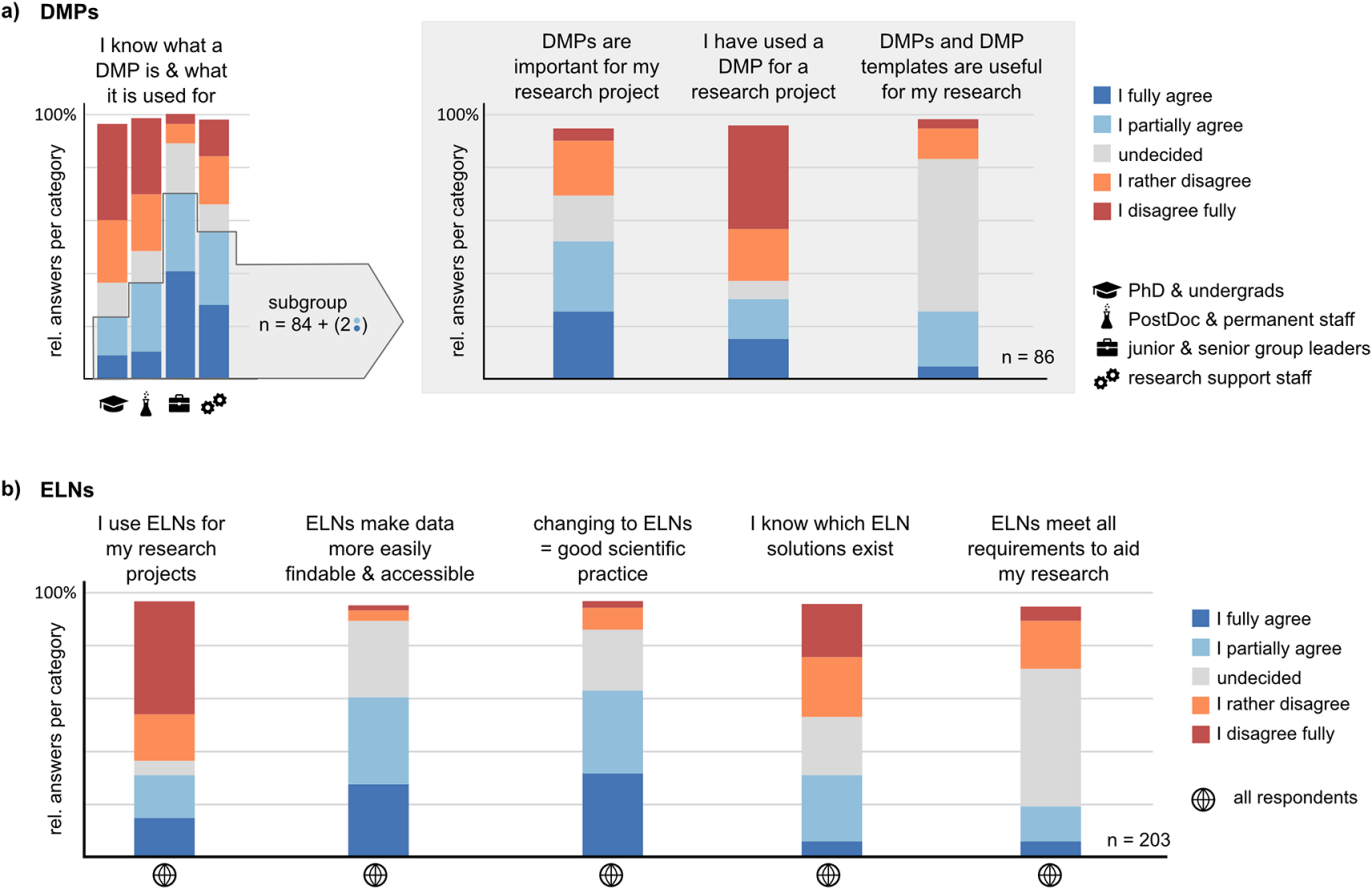
Knowledge and use of Data Management Plans (DMPs) and Electronic Lab Notebooks (ELNs). a) Answers of participants from the four career level groups (undergraduate and PhD students, n = 55; postdoctoral and permanent researchers, n = 66; junior and senior group leaders/professors, n = 27; research support staff, n = 50) to the statement “I know what a DMP is and what it is used for”. The answers to the right-hand statements (grey background) are only shown for participants who stated to know what a DMP is (84 respondents from career level groups, 2 respondents who stated “Company” and “Consultant” as their career level). b) Answers of all participants to the indicated statements about ELNs.

Data handling can be assisted by planning the necessary management steps throughout the data life cycle and by documenting experiments in electronic rather than paper-based lab notebooks. We asked about the use and knowledge of DMPs and ELNs in general. Junior and senior group leaders had the highest relative fraction of respondents stating to know what a DMP is and what it is used for, followed by the research support group ( Figure 6a). However, among all respondents only 86 answered to this question with “I fully agree” or “I partially agree” (84 from the four career level groups and two not included in the subgroups). Due to the low absolute numbers of respondents stating to know what a DMP is in some career level groups (e.g., 13 persons of the undergraduate & PhD students, and 19 persons of the junior and senior group leaders) we analyzed the following questions cumulatively for all 86 respondents ( Figure 6a and Suppl. Figure 9a ^35^; for an analysis of individual groups, see Suppl. Figure 9b ^35^). Many have not previously used a DMP, but overall, these 86 participants agree that DMPs are valuable for their work. However, they are somewhat undecided about the quality of DMP guidelines and templates, indicating that method- and discipline-specific templates for the creation of DMPs are missing. Existing efforts to provide DMP templates, online guides e.g., Open Science Framework Guides, DMP tools (e.g., RDMO ^19^), or DMP guidelines (e.g., Ref. 49) should therefore be improved to better address specific user needs. As the requirements of FAIR data handling should be considered before the start of a research project, DMPs might serve as valuable tools, and several funding agencies demand DMPs as part of grant applications for third-party funding (e.g., German Research Foundation, DFG, European Research Council, ERC).

Only about one-third of the participants use an ELN, which is a similarly low fraction as the fraction of core facilities using ELNs or Laboratory Information Management Systems (LIMS) reported in a 2020 survey. ^33^ Yet, participants in our survey generally regard ELNs as valuable for data management and to facilitate good scientific practice. At the same time, they are unsure about which solutions exist and if they are suitable for their research ( Figure 6b and Suppl. Figure 10 ^35^). In total, respondents endorse the use and value of both DMPs and ELNs, but the practical experience with these tools is rather limited. Use cases and best practice examples might be required to improve the adoption of DMPs and ELNs by researchers.

### RDM literacy is regarded as valuable but time-consuming and is not part of academic education

**Figure 7.**
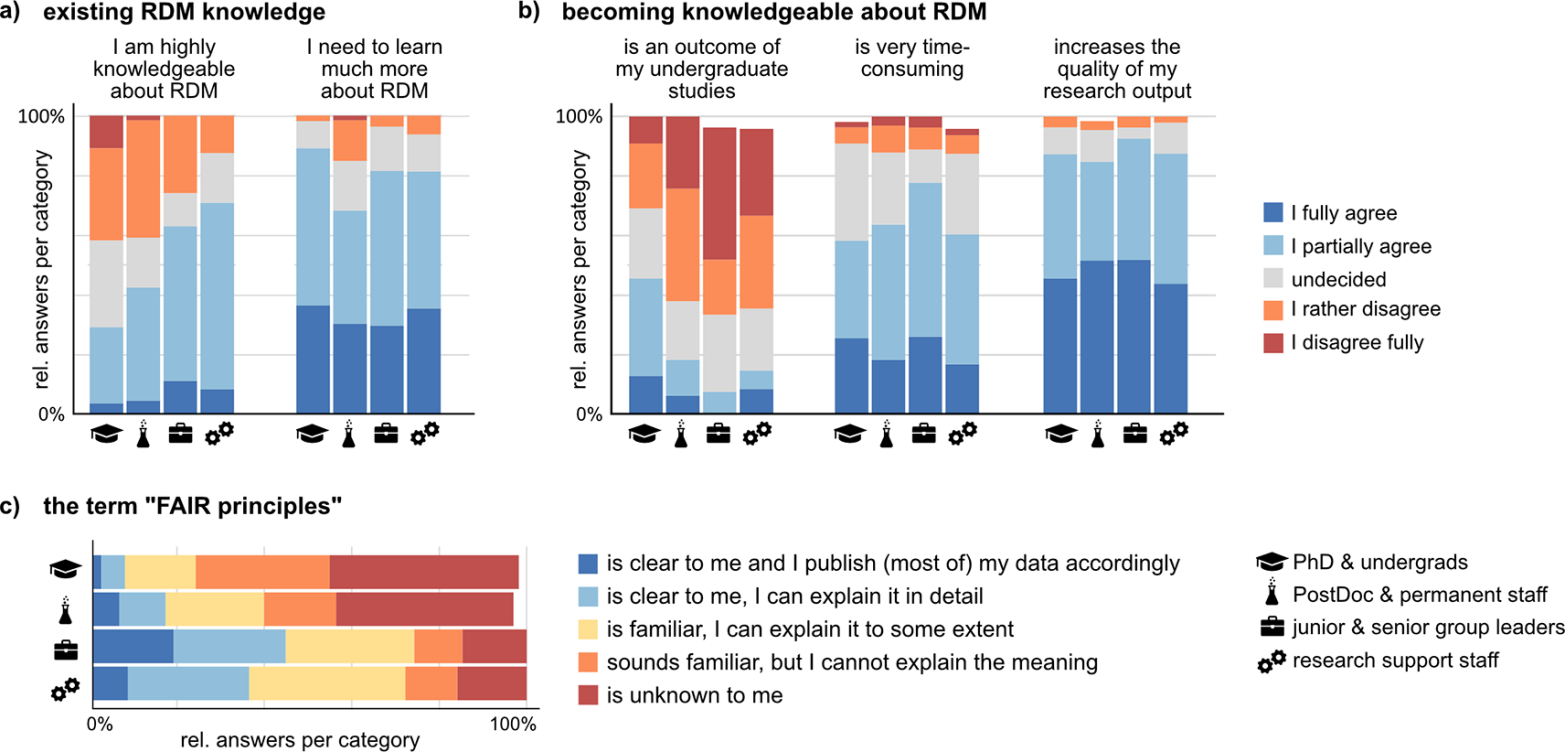
RDM knowledge and the FAIR (finable, accessible, interoperable, reusable) principles. a, b) Respondents provided their opinion about presented statements on a five-item scale. See Suppl. Figure 11 for the full set of questions. Shown are the relative fraction per answer item in each group (undergraduate and PhD student, n = 55; Postdoctoral and permanent researchers, n = 66; Junior and senior group leaders, n = 27; Research support and facility staff, n = 50). c) Respondents were asked about their adoption and knowledge of the FAIR principles on the indicated five-item answer scale.

To learn more about the education, state of knowledge, and motivation of respondents to become proficient in research data management, we asked participants about their opinion on statements about RDM literacy. Between 30% and 70% of respondents judge themselves as highly knowledgeable about RDM, with the highest fraction found in the research support group. Yet, all groups report a high demand to learn more about RDM in their field ( Figure 7a). About half of the respondents declare to handle their data according to community standards, and about half say that they handle data according to their own individual standards. However, 20% of the respondents agree fully or partially to both, using own as well as community standards at the same time. Accordingly, there is only a weak negative correlation between the agreement to the two statements (Suppl. Figure 11 ^35^). All groups agree that becoming knowledgeable about RDM is valuable for their research but very time-consuming ( Figure 7b). Interestingly, almost 50% of PhD and undergraduate students state that becoming knowledgeable about RDM is an outcome of their education during undergraduate studies, a markedly higher proportion than in the other groups ( Figure 7b). This result suggests that RDM has started to become accessible to young researchers via university curricula. At the same time, quite surprisingly, the same group of young researchers at the undergraduate/PhD level but also at the Postdoc-level has the lowest fractions of respondents who adhere to, know, or at least are familiar with the FAIR principles ( Figure 7c). On average, about 9% of respondents state to publish their data according to the FAIR principles, and about 18% know about the FAIR principles in detail. This result indicates a more limited adoption of the FAIR principles than suggested by the 2021 State of Open Data survey, ^50^ which contained the highest percentage of respondents who state that their data is “very much” or “somewhat” FAIR compliant (54%) since the question was first asked. The discrepancy between statements about general RDM knowledge and understanding the FAIR principles indicates that albeit the awareness about RDM is increasing, the concepts are far from being clear.

### Despite a high willingness, data sharing and reuse are rarely established in practice due to technical hurdles, lack of guidelines, and insecurity about legal aspects

**Figure 8.**
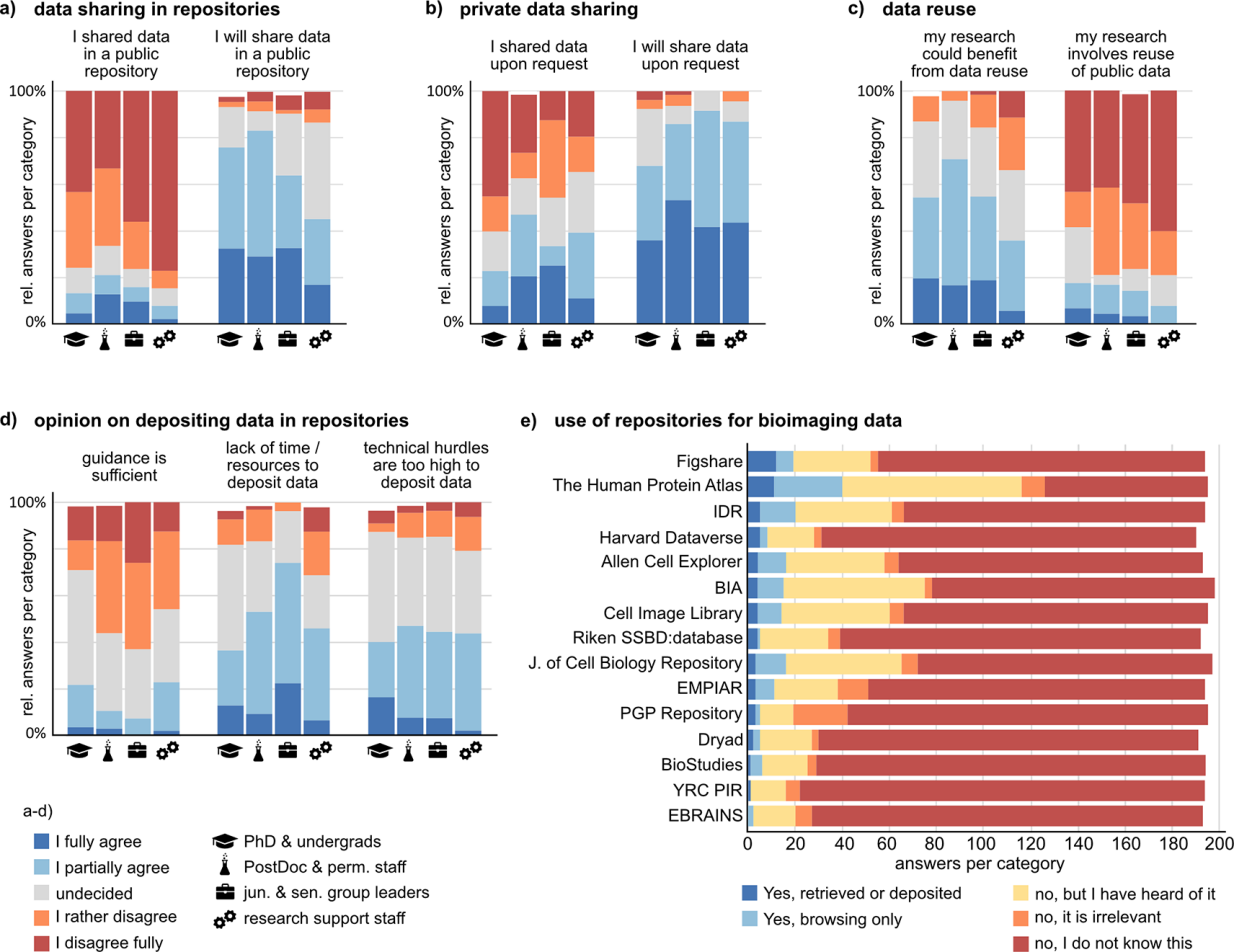
Experience with and opinion about bioimage data sharing. a, b, c) Shortened statements about private or public data sharing and data reuse. Shown are the relative answer distributions of the five agreement levels for each of the analyzed groups (undergraduate and PhD students, n = 55; Postdoctoral and permanent researchers, n = 66; junior and senior group leaders/professors, n = 27; Research support staff, n = 48). d) Opinions on three statements about repositories. e) Knowledge and use of preselected data repositories including bioimaging-specific, research-area-specific, and generic repositories. See Suppl. Figure 12 ^35^ for the full set of questions corresponding to this figure. Abbreviations: BIA BioImage Archive; EMPIAR Electron Microscopy Public Image Archive; IDR Image Data Resource; PGP Plant Genomic and Phenomics; YRC PIR Yeast Research Center Public Image Repository.

An important goal of fostering RDM standards is to increase the sustainability of the scientific system by enabling (public) data sharing, access to data, and reuse. At the same time, FAIR-managed data can facilitate trust in scientific findings as it enhances the ability to understand and reproduce experiments and their results. In this survey, we asked the participants about their practices and opinion on bioimage data sharing and reuse. On average, about 50% of the participants partially or fully agree that their bioimaging data might be valuable for answering (parts of) other researchers’ questions (Suppl. Figure 12 ^35^). However, there is a marked difference between the stated willingness to share data privately upon request or publicly in a repository and the actual practice of sharing data ( Figure 8a and b). Moreover, many researchers agree that reusing publicly available bioimage data could benefit their own research. Still, only a low fraction states to involve image data reuse in their work ( Figure 8c).

A prerequisite for public sharing and reuse of data are public repositories enabling searching and accessing published data. We presented a preselected list of data repositories, including bioimaging-specific, research-area-specific and generic repositories, asking if and how participants have used one or more of these repositories ( Figure 8e). The majority of repositories was unknown to more than 60% of participants. The lowest relative fraction of “I don’t know this”-answers was found in the research support group, and the highest in the undergraduate and PhD students group. The best known and most often actively used repository was The Human Protein Atlas. ^47^ The preselected list also included one discontinued repository, the Journal of Cell Biology repository (JCB Data Viewer). Some of the listed repositories are specific to a single research discipline and hence are not relevant for all respondents. However, even bioimage-data-specific or generic repositories were not broadly known. Further repositories that participants entered in a free-text field included Zenodo (5x), an own university repository (4x), MorphDBase (2x), OMERO (2x), GitHub, BonaRes repository, The Protein Data Bank, NTU Dataverse, Metaspace, nanotomy.org, and Genepaint.

To find out about possible hurdles with respect to public data sharing, we polled the opinion towards various statements about repositories ( Figure 8d, and Suppl. Figure 12 ^35^). Most prominently, insufficient guidance towards appropriate repositories, technical hurdles, and lack of time or resources were declared as impediments to repository-based data sharing. Standard operating procedures on how to submit data to repositories, including information on the legal framework of data sharing and licensing could improve the practice of data sharing and ultimately allow higher reuse of published bioimaging data for novel research questions.

## Conclusions

In this survey, we investigated the state-of-the-art of bioimage research data management among bioimaging scientists and research support staff, mainly in Germany but also beyond, since almost one-third of respondents are located outside of Germany. The survey results give a valuable snapshot of the current practices in bioimaging RDM, including the perspectives from many research disciplines and career levels. Thus, the survey answers provide a resource to design RDM measures for bioimaging, taking the different needs of different user groups into account. Results about image analysis and the use of ELNs match with results conducted in similar previous surveys, suggesting a good overall validity of the designed questions. However, the relatively small number of participants and the main survey announcement channels bear a risk of bias towards community members closely associated with GerBI or NFDI4BIOIMAGE. The representativeness of the results might hence be limited to respondents with an above-average interest in microscopy. Follow-up surveys should be designed to include a broader representation of microscopy users taking into account the connection to FAIR data needs for different disciplines and research techniques. The survey data is also limited with respect to interpretations about the RDM requirements for advanced imaging modalities that are less common than, e.g., confocal microscopy, or the needs of particular user groups, e.g., pure data analysists as opposed to wet lab scientists. The survey results show that there is a demand for more knowledge about bioimage RDM but also for generic RDM principles and concepts (i.e., the FAIR principles, the research data life cycle, or data management plans). Where they are known, these principles are well acknowledged and endorsed but their practical implementation in the everyday work of bioimaging scientists is clearly lagging behind. However, respondents could have different interpretations of terms like “DMP”, “data management system” or even “research data management” in general. For example, respondents might see a DMP as a non-formalized short document, or maybe as a comprehensive form that needs to be filled out. Here, no definitions were offered prior to asking the question. For clarity, the authors interpret “research data management” as any activity dedicated to organized handling of any type of physical or digital data associated with the conducted research. A “data management system” is regarded as a software- or hardware-based technical installation to fulfill (aspects of) research data management. A DMP is interpreted as any written, formalized or non-formalized planning of data management activities during and after the research is conducted. As shown by the survey results and further highlighted by free-text comments (Ext. Data 1 ^35^), the needs of individual researchers or support staff range from a basic understanding of RDM principles over infrastructural problems to specific issues with proprietary file types and storage servers.

### Future perspective

**Figure 9.**
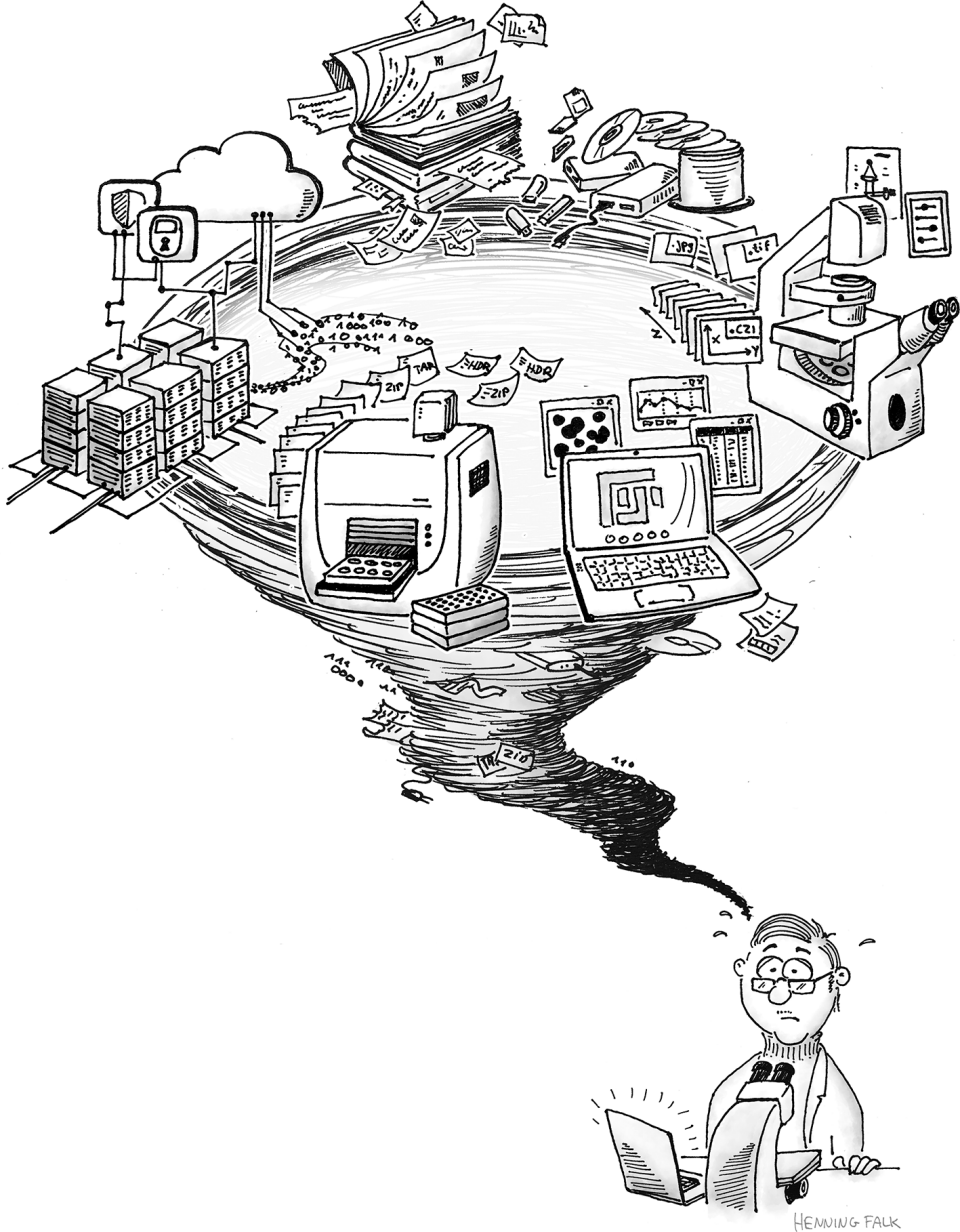
Proper handling of large-scale, complex data as frequently acquired in bioimaging is a challenge for researchers, data providers, and data users. Targeted measures must rely on a firm knowledge of the community perspective and its needs to transform the research data management whirlwind into a well-managed bioimage data life cycle (cartoon produced by Henning Falk for this article and published with permission).

How can interoperable standards cover the various levels of complexity of bioimaging and its diverse applications in different research fields? These challenges are being tackled by multiple initiatives that have, for example, proposed a set of standard file formats for bioimaging, ^10^^,^^27^ a tiered system for metadata specifications, ^51^ and have created imaging-specific repositories. ^23^^–^^25^ For example, novel tools for metadata annotation ^28^^,^^29^^,^^52^ are now available to be tested and refined in use case scenarios. The survey results show that only a small percentage uses these and harvests their benefits. The main question is: How can the already existing developments be integrated into everyday research? This requires best practice examples and iterative testing and refinement of the tools, tailored training material, and, additionally, guidelines specific for bioimaging. For example, how do users annotate bioimaging metadata in practice, and which metadata should be recorded for which bioimaging experiment? And how can researchers stay abreast of the developments and solutions produced by the international community? In other words, how can one most effectively help to turn the whirlwind of concepts, tools, and guidelines on the topic of research data management into a well-established and easy-to-adhere-to research data life cycle for bioimaging researchers ( Figure 9)? This survey also shows that scientific core facilities are of prime importance for education and training in scientific methods. As an integral part of the research infrastructure, bioimaging core facilities are in an excellent position to facilitate and promote data FAIRification. They are often essential members in third-party-funded research programs (e.g., collaborative research centers in Germany), and they are confronted with the needs of users from many research disciplines on a daily basis. Core facilities combine scientific knowledge with technical expertise and practical experience, often including support in bioimage analysis and statistics. Moreover, they interact with IT services at local institutions, and, importantly, are well-connected in networks both at the national and the international level.

A prerequisite to fulfill this potential for the benefit of bioimage data FAIRification is the availability of dedicated funding. Members of GerBI-GMB have successfully engaged in several activities to contribute to novel solutions for bioimaging RDM, e.g., the abovementioned RDM4mic group or the DFG-funded small-scale infrastructure project on OMERO ( I3D:bio, Information Infrastructure for BioImage Data). Currently, GerBI-GMB participates in the consortium initiative NFDI4BIOIMAGE which has applied for funding in the third call of the NFDI.

Two types of future actions appear to be mainly required: first, training and education must be available at all levels, from basic to advanced, for all stakeholders. Resources must include clear hands-on use case examples of how to apply FAIR principles in practice to foster adoption by users. Second, to do so, tools, guidelines, and standard operating procedures must be developed according to user-specific needs, tested and refined iteratively, and integrated into the wider international RDM landscape. Our survey exposes the marked gap between the willingness of the community to share and reuse bioimaging data versus its ability to do so, and outlines necessary actions to fill this gap, thereby contributing to the ongoing efforts for the FAIRification of research data across disciplines.

## Author contributions

C.S. and J.H. drafted the questionnaire, managed the project and the data, analyzed the data, created the figures and wrote the manuscript. J.M. contributed to the questionnaire and analysis and refined the manuscript and figures. C.M., contributed to the questionnaire and reviewed the manuscript. S.W.P. and E.F.M. supervised the project, refined the questionnaire, contributed to the manuscript and refined it.

## Data availability

### Underlying data

Zenodo: Research data management for bioimaging: the 2021 NFDI4BIOIMAGE community survey - Extended Data 3 - Raw Data survey entries, https://doi.org/10.5281/zenodo.6504466. ^37^

This project contains the underlying original data in csv format.

Zenodo: Research data management for bioimaging: the 2021 NFDI4BIOIMAGE community survey - Extended Data 4 - Analysis Data Sheet, https://doi.org/10.5281/zenodo.7082609. ^38^

This project contains the xlsx-sheet containing comments, provenance on QC, raw graphs, and subgroups.

### Extended data

Zenodo: Research data management for bioimaging: the 2021 NFDI4BIOIMAGE community survey - Extended Data 1 - Supplementary Information and Supplementary Figures, https://doi.org/10.5281/zenodo.7082514. ^35^

This project contains supplementary information and supplementary figures containing details on the quality control procedure, the questionnaire logic, free-text comments, and supplementary figures.

Zenodo: Research data management for bioimaging: the 2021 NFDI4BIOIMAGE community survey - Extended Data 2 – Questionnaire, https://doi.org/10.5281/zenodo.6504207. ^36^

This project contains the questionnaire with question identifiers and comments on conditional logic.

Data are available under the terms of the Creative Commons Attribution 4.0 International license (CC-BY 4.0).
