# Supplementary material for: Research data management for bioimaging: the 2021 NFDI4BIOIMAGE community survey
Source: F1000Res. 2022 Sep 20;11:638. Originally published 2022 Jun 10. [Version 2] doi: 10.12688/f1000research.121714.2 (PMC9641114; doi:10.12688/f1000research.121714.2)
Supplement: Peer review history

# Peer review: Reviewer response for version 1

**DOI:** 10.5256/f1000research.133609.r140332

**Published:** 2022-08-08

**Authors:** Marjolijn Mertz, Rolf Harkes

**Affiliations:** 1 Bioimaging Facility, The Netherlands Cancer Institute, Amsterdam, The Netherlands

Advances in bioimaging techniques have allowed for the acquisition of larger and more complex image datasets. Image processing and analysis workflows can now run fully automated or with (minimal) intervention of the researcher. These developments call for structured data infrastructures and storage according to the FAIR principles (findable, accessible, interoperable and reproducible). Requirements might differ between institutions and/or core-facilities due to size, available technologies and user demands. Nevertheless, we can learn from each other in 'how to’ optimize data management, image analysis infrastructure and metadata structure by exchanging experiences and insights.

 In this extensive community survey Schmidt et al. have done an excellent job in mapping out the current status and needs for research data management (RDM) in the bioimaging community. The survey results give insight in all parts of the bioimaging research data life cycle, with questions covering acquisition techniques, image analysis and data management.

 The survey was launched online via the NFDI4BIOIMAGE website on June, 1st, 2021 and closed July, 21st, 2021. The methodology is well described and survey questions are available in the supplemental data.

 A total of 203 completed survey responses are included in the analysis, with most respondents working in Germany (143), the other 60 were distributed equally over EU and non-EU countries. The majority of participants work at a university or non-profit institute. The authors categorized respondents by career level (researchers, undergrads, PI's and facility staff) and/or experience level. It is clear that FAIR data management is seen as necessary at all levels. The survey identifies RDM knowledge, consensus on what standards to use and IT infrastructure as areas that need improvement to enable the successful implementation of RDM in bioimaging data.

 The authors recommend two actions required to achieve these improvements. First, education on RDM of all levels and secondly development and implementation of RDM tools, guidelines and SOPs. Schmidt et al. foresee an important role for core facility staff because of their knowledge in all aspects of the bioimaging workflow, expertise in teaching scientific methods and close contacts with the IT department.

General comments:

- The survey has been aimed mainly at the community members of the organizations that the authors are affiliated with. This is reflected in the frequent usage of ImageJ and Omero by the respondents. The authors already acknowledge the relatively small number of participants might bear some risk of bias towards community members closely associated with GerBI or NFDI4BIOIMAGE. RDM and FAIR data are of great importance, not only to the bioimaging community, but to other communities as well. Therefore the implementation of RDM tools within an institute will have to also include other –omics and microscopy data users that are outside of the NFDI4BIOIMAGE community

Minor issues:

- Schmidt et al. point out their career level response is heterogeneous with respect to career and experience level, but do not mention if the distribution is representative of the academic population involved in bioimage analysis. For example, in our experience there are substantially more PhD students that work on bioimage analysis as compared to facility staff members. However, they are nearly equally represented in the survey. Therefore we recommend in figure 6a “I know what a DMP is & what it is used for” to include the distribution over the four subgroups. The same way it has been done in figure 5, 7 and 8.
- The survey uses the terms Data Management Systems, Research Data Management (RDM) and Data Management Plan (DMP). In our experience especially DMP has a broad meaning in the community. From a few simple rules about where to store/backup data, to a complete data lifecycle plan. We agree that excluding users that don’t know what a DMP is from further questions about DMPs is a good approach. However, we think it would have been better to explain briefly what was meant by the authors with the three terms and their relation to each other, and only then ask the user if the information about DMPs was new to them. We recommend a very brief explanation of the three terms in the paper to educate the readers and advise this course of action in a potential follow-up survey.

Is the work clearly and accurately presented and does it cite the current literature?

Yes

If applicable, is the statistical analysis and its interpretation appropriate?

I cannot comment. A qualified statistician is required.

Are all the source data underlying the results available to ensure full reproducibility?

Yes

Is the study design appropriate and is the work technically sound?

Yes

Are the conclusions drawn adequately supported by the results?

Yes

Are sufficient details of methods and analysis provided to allow replication by others?

Yes

Reviewer Expertise:

Light microscopy,  Image processing & analysis,  Data management, BioImaging data infrastructure

We confirm that we have read this submission and believe that we have an appropriate level of expertise to confirm that it is of an acceptable scientific standard.

# Response

**Published:** 2022-09-15

**Authors:** Christian Schmidt

**Affiliations:** German Cancer Research Center, Germany

We thank the reviewers for taking the time to asses our manuscript. We addressed all concerns, a detailed answer is in line with the reviewer‘s comments below. 

**Review letter**

 Advances in bioimaging techniques have allowed for the acquisition of larger and more complex image datasets. Image processing and analysis workflows can now run fully automated or with (minimal) intervention of the researcher. These developments call for structured data infrastructures and storage according to the FAIR principles (findable, accessible, interoperable and reproducible). Requirements might differ between institutions and/or core-facilities due to size, available technologies and user demands. Nevertheless, we can learn from each other in 'how to’ optimize data management, image analysis infrastructure and metadata structure by exchanging experiences and insights.

 In this extensive community survey Schmidt et al. have done an excellent job in mapping out the current status and needs for research data management (RDM) in the bioimaging community. The survey results give insight in all parts of the bioimaging research data life cycle, with questions covering acquisition techniques, image analysis and data management.

 The survey was launched online via the NFDI4BIOIMAGE website on June, 1st, 2021 and closed July, 21st, 2021. The methodology is well described and survey questions are available in the supplemental data.

 A total of 203 completed survey responses are included in the analysis, with most respondents working in Germany (143), the other 60 were distributed equally over EU and non-EU countries. The majority of participants work at a university or non-profit institute. The authors categorized respondents by career level (researchers, undergrads, PI's and facility staff) and/or experience level. It is clear that FAIR data management is seen as necessary at all levels. The survey identifies RDM knowledge, consensus on what standards to use and IT infrastructure as areas that need improvement to enable the successful implementation of RDM in bioimaging data.

 The authors recommend two actions required to achieve these improvements. First, education on RDM of all levels and secondly development and implementation of RDM tools, guidelines and SOPs. Schmidt et al. foresee an important role for core facility staff because of their knowledge in all aspects of the bioimaging workflow, expertise in teaching scientific methods and close contacts with the IT department.

**Answer**

 We thank the reviewers for their positive comments and their assessment of the manuscript. The reviewers described the outline and the main points of the manuscript to a high level of detail. 

**General comments:**

- The survey has been aimed mainly at the community members of the organizations that the authors are affiliated with. This is reflected in the frequent usage of ImageJ and Omero by the respondents. The authors already acknowledge the relatively small number of participants might bear some risk of bias towards community members closely associated with GerBI or NFDI4BIOIMAGE. RDM and FAIR data are of great importance, not only to the bioimaging community, but to other communities as well. Therefore the implementation of RDM tools within an institute will have to also include other –omics and microscopy data users that are outside of the NFDI4BIOIMAGE community. 

**Answer:**

 We agree with the reviewers that a connection and collaboration between different techniques and disciplines is necessary to achieve FAIR data management in science. In this study, we tried to include respondents beyond the bioimaging community by forwarding the survey to other NFDI consortia (both, already funded and in preparation). From the survey questions asked, however, we cannot estimate how many respondents acquire -omics data in addition to bioimaging data. We see the added value for future studies and plan to include more questions in this direction in future surveys. We thank the reviewers for this suggestion. We added the following text to the “Conclusions” section:

 “The representativeness of the results might hence be limited to respondents with an above-average interest in microscopy. Follow-up surveys should be designed to include a broader representation of microscopy users taking into account the connection to FAIR data needs for different disciplines and research techniques.”

**Minor issues:**

- Schmidt et al. point out their career level response is heterogeneous with respect to career and experience level, but do not mention if the distribution is representative of the academic population involved in bioimage analysis. For example, in our experience there are substantially more PhD students that work on bioimage analysis as compared to facility staff members. However, they are nearly equally represented in the survey. Therefore we recommend in figure 6a “I know what a DMP is & what it is used for” to include the distribution over the four subgroups. The same way it has been done in figure 5, 7 and 8.

**Answer**

 We thank the reviewers for this comment. We have included the distribution analysis across subgroups in Figure 6a for the first question “I know what a DMP is & what it is used for”. The same analysis was performed for the remaining panels of Figure 6a and added to Suppl. Figure 9b (Ext. Data 1).

 The text referring to figure 6 now reads as follows:

 “Junior and senior group leaders had the highest relative fraction of respondents stating to know what a DMP is and what it is used for, followed by the research support group (Figure 6 a). However, among all respondents only 86 respondents answered to this question with “I fully agree” or “I partially agree” (84 from the four career level groups and two not included in the subgroups). stated to know what a DMP is and what it is used for, but most of them have not used a DMP before ( Figure 6a). Due to the low absolute numbers of respondents stating to know what a DMP is in some career level groups (e.g., 13 persons of the undergraduate & PhD students, and 19 persons of the junior and senior group leaders) we analyzed the following questions cumulatively for all 86 respondents ( Figure 6a and Suppl. Figure 9a; for an analysis of individual groups, see Suppl. Figure 9b).”

 And the figure legend:

 “Answers of all participants from the four career level groups (undergraduate and PhD students, n = 55; postdoctoral and permanent researchers, n = 66; junior and senior group leaders/professors, n = 27; research support staff, n = 50) to the indicated statement “I know what a DMP is and what it is used for”. The answers to the right-hand statements (grey background) are only shown for participants who stated to know what a DMP is (84 respondents from career level groups, 2 respondents who stated “Company” and “Consultant” as their career level).“

- The survey uses the terms Data Management Systems, Research Data Management (RDM) and Data Management Plan (DMP). In our experience especially DMP has a broad meaning in the community. From a few simple rules about where to store/backup data, to a complete data lifecycle plan. We agree that excluding users that don’t know what a DMP is from further questions about DMPs is a good approach. However, we think it would have been better to explain briefly what was meant by the authors with the three terms and their relation to each other, and only then ask the user if the information about DMPs was new to them. We recommend a very brief explanation of the three terms in the paper to educate the readers and advise this course of action in a potential follow-up survey.

**Answer:**

 We thank the reviewers for this suggestions. We have included the authors' understanding of the terms DMP, RDM and data management system in the “Conclusions” section. It now reads as follows (“Conclusions”, 2nd paragraph):

 “However, respondents could have different interpretations of terms like “DMP”, “data management system” or even “research data management” in general. For example, respondents might see a DMP as a non-formalized short document, or maybe as a comprehensive form that needs to be filled out. Here, no definitions were offered prior to asking the question. For clarity, the authors interpret “research data management” as any activity dedicated to organized handling of any type of physical or digital data associated with the conducted research. A “data management system” is regarded as a software- or hardware-based technical installation to fulfil (aspects of) research data management. A DMP is interpreted as any written, formalized or non-formalized planning of data management activities during and after the research is conducted.“

# Peer review: Reviewer response for version 1

**DOI:** 10.5256/f1000research.133609.r140961

**Published:** 2022-06-21

**Authors:** Oliver Biehlmaier

**Affiliations:** 1 IMCF Imaging Core Facility Biozentrum, Biozentrum of the University of Basel, Basel, Switzerland

In recent years, the move towards research data management according to the F.A.I.R principles has gained momentum tremendously. The motivation for this important development can be traced back to funding agencies, university policies, and most recently educational developments in individual institutions, that empower the implementation of respective infrastructures and personnel, like data stewards. The paper by Schmidt, Hanne et al adds numbers to these developments with the results of a survey conducted in June/July 2021 among more than 200 bioimaging users. The demographics of the respondents are centered on Germany, the EU and non-EU countries. It therefore is a valuable complement to other surveys focused on other geographic regions. The survey, as published in the supplementary information, is comprehensive, yet the authors were able to collect over 200 useable response sets with a drop-out rate of 11.7%, which in itself may be seen as an indication of the topic’s relevance and timeliness.

 The dataset obtained through the survey and made available as supplementary information allows a number of analyses on different subpopulations and their usage and unmet needs in image analysis and data management. Many of the most crucial questions have been asked in this paper e.g. Which imaging techniques are the most used? What is the most time consuming? How do you approach image analysis? Which repositories are in use? Is the importance of metadata clear?

 The most interesting of these analyses are presented in the paper, with further analyses relegated to supplementary figures. Beyond the analyses offered in the paper, we would like to specifically point out the underlying data set as a carefully designed and curated resource that no doubt can be used to answer subsequent questions.

 The conclusions drawn from the questionnaire not only clearly show the important role of core facilities in the guidance of users towards appropriate data management strategies, but also delineate the major obstacles: while the necessity for data management systems is obvious, these systems are not yet put into place everywhere. Moreover, respondents identify the need to become more expert in research data management regards, and often feel left alone without guidance. This is a clear call for increased training in this topical area. To facilitate proper annotation, curation, and deposition, new tools must be developed that lower technical hurdles to appropriate data deposition. 

 Minor issues:

- Recruitment of the respondents: Established channels (web sites, discussion forums, conference / society mailing lists) for scientists and facility staff involved and interested in image analysis were used to recruit survey respondents. Very likely, this sampling of microscopy users is biased towards researchers with particular interest in the topic and motivation. However, this bias is probably unavoidable when recruiting volunteers in this manner. It would be good if this point could be mentioned more clearly in the methodology.
- Use and experience of data management plans is discussed and shown in Fig 6. The finding of <50% of respondents being familiar with a DMP is a bit surprising, given that DMPs are nowadays required for many major funding instruments in Europe. It would be interesting to resolve the responses by career stage, e.g. do group leader and facility staff know about them, but more junior scientists do not?
- Questions regarding the willingness of respondents to fulfil data management requirements (as discussed e.g. in fig. 6, 7 and 8, as well as supplemental figure 11a: In political polling, it has been demonstrated that asking voters about their intentions, rather than their expectations, leads to worse outcome predictions, likely not only because respondents are drawn towards describing own behavior with the perceived politically appropriate option, they also tend to include the prevailing opinions of their social circle when asked about expectations. For instance, see Murr, A., Stegmaier, M., & Lewis-Beck, M. (2021). Vote Expectations Versus Vote Intentions: Rival Forecasting Strategies. *British Journal of Political Science,* *51*(1), 60-67.
  For the questions about views and behaviour regarding RDM, expectation questions could have added another perspective, and perhaps helped to mitigate the bias inherent in the recruitment. We realize, however, that this can't be added post-hoc and is intended as a remark for future studies.

Is the work clearly and accurately presented and does it cite the current literature?

Yes

If applicable, is the statistical analysis and its interpretation appropriate?

Not applicable

Are all the source data underlying the results available to ensure full reproducibility?

Yes

Is the study design appropriate and is the work technically sound?

Yes

Are the conclusions drawn adequately supported by the results?

Yes

Are sufficient details of methods and analysis provided to allow replication by others?

Yes

Reviewer Expertise:

Biology, light and electron microscopy, image processing, bioimage data repositories, data management, metadata

I confirm that I have read this submission and believe that I have an appropriate level of expertise to confirm that it is of an acceptable scientific standard.

## References

[1] : Vote Expectations Versus Vote Intentions: Rival Forecasting Strategies. *British Journal of Political Science* .2021;51(1) : 10.1017/S0007123419000061 60-67 10.1017/S0007123419000061

# Response

**Published:** 2022-09-15

**Authors:** Christian Schmidt

**Affiliations:** German Cancer Research Center, Germany

We would like to thank the reviewer for taking the time to read and evaluate our manuscript, and to write a review letter. We are grateful for the overall positive feedback on our work. The reviewer raises minor issues to improve the quality of the presented work or to be taken into account for follow-up surveys. We highly appreciate this input. We have addressed all reviewer comments and would like to reply to the review letter point by point in more detail.

**Review letter:**

 In recent years, the move towards research data management according to the F.A.I.R principles has gained momentum tremendously. The motivation for this important development can be traced back to funding agencies, university policies, and most recently educational developments in individual institutions, that empower the implementation of respective infrastructures and personnel, like data stewards. The paper by Schmidt, Hanne et al adds numbers to these developments with the results of a survey conducted in June/July 2021 among more than 200 bioimaging users. The demographics of the respondents are centered on Germany, the EU and non-EU countries. It therefore is a valuable complement to other surveys focused on other geographic regions. The survey, as published in the supplementary information, is comprehensive, yet the authors were able to collect over 200 useable response sets with a drop-out rate of 11.7%, which in itself may be seen as an indication of the topic’s relevance and timeliness.

 The dataset obtained through the survey and made available as supplementary information allows a number of analyses on different subpopulations and their usage and unmet needs in image analysis and data management. Many of the most crucial questions have been asked in this paper e.g. Which imaging techniques are the most used? What is the most time consuming? How do you approach image analysis? Which repositories are in use? Is the importance of metadata clear?

 The most interesting of these analyses are presented in the paper, with further analyses relegated to supplementary figures. Beyond the analyses offered in the paper, we would like to specifically point out the underlying data set as a carefully designed and curated resource that no doubt can be used to answer subsequent questions.

 The conclusions drawn from the questionnaire not only clearly show the important role of core facilities in the guidance of users towards appropriate data management strategies, but also delineate the major obstacles: while the necessity for data management systems is obvious, these systems are not yet put into place everywhere. Moreover, respondents identify the need to become more expert in research data management regards, and often feel left alone without guidance. This is a clear call for increased training in this topical area. To facilitate proper annotation, curation, and deposition, new tools must be developed that lower technical hurdles to appropriate data deposition.

**Answer:**

 We thank the reviewers for the summary and highlighting of important aspects of our work. We appreciate that the reviewer agrees with the most important findings and conclusions of our study.

**Minor issue 1**

 Recruitment of the respondents: Established channels (web sites, discussion forums, conference / society mailing lists) for scientists and facility staff involved and interested in image analysis were used to recruit survey respondents. Very likely, this sampling of microscopy users is biased towards researchers with particular interest in the topic and motivation. However, this bias is probably unavoidable when recruiting volunteers in this manner. It would be good if this point could be mentioned more clearly in the methodology.

**Answer:**

 We agree that the data has an inherent bias towards answers from participants with an above-average interest in bioimaging techniques. Most of our communication channels for participant recruitment are prominently used by the bioimaging community. While for this survey this community was our intended main target group, we tried to open up more widely to microscopy users in all areas of research by advertising the survey via the speakers of other NFDI consortia, whose communities cover a wide range of disciplines, and by public advertising on Twitter. However, only about ~17 % of the respondents using bioimaging techniques use them less than at least once per week (Suppl. Fig. 3c). Including the perspectives of researchers who use microscopes rather occasionally for specific questions more extensively in follow-up work could increase the representativeness of the data for microscopy users overall. To point out this bias more transparently we rephrased the methods sections accordingly and extend the discussion of this point in the “Conclusions” section.

 Methods section: „The link was shared via the newsletter of German BioImaging – Gesellschaft für Mikroskopie und Bildanalyse e.V. (GerBI-GMB), which addresses researchers, companies, core facilities and institutes within GerBI-GMB, and their respective users. Additionally, the link to participate in the survey was shared with participants at the ELMI conference 2021, on the NFDI4BIOIMAGE website, via the Confocal Mailing List, and at the Euro-BioImaging Virtual Pub on June 4th, 2021. To mitigate a sole bias towards members of the bioimaging community closely associated with GerBI-GMB, we asked the spokespersons of established and planned NFDI consortia to distribute the invitation within their communities. These consortia cover a wide range of scientific disciplines. The survey was furthermore announced in three posts on Twitter.“

 Conclusions section: „However, the relatively small number of participants and the main survey announcement channels bear a risk of bias towards community members closely associated with GerBI or NFDI4BIOIMAGE. The representativeness of the results might hence be limited to respondents with an above-average interest in microscopy. Follow-up surveys should be designed to include a broader representation of microscopy users taking into account the connection to FAIR data needs for different disciplines and research techniques.“

**Minor issue 2:**

 Use and experience of data management plans is discussed and shown in Fig 6. The finding of <50% of respondents being familiar with a DMP is a bit surprising, given that DMPs are nowadays required for many major funding instruments in Europe. It would be interesting to resolve the responses by career stage, e.g. do group leader and facility staff know about them, but more junior scientists do not?

**Answer:**

 We thank the reviewer for pointing out this interesting aspect. We analyzed the answers to this question per career-level groups (84 out of 86, since two respondents stated their career levels to be “company” and “consultant” in the free text field). As hypothesized by the reviewer, we observe that the relative fractions of respondents stating to know what a DMP is are highest among the junior/senior group leaders (~ 70 %) and among research support staff (~ 56 %). In contrast, only ~ 36 % (post-doc & permanent researchers) and ~ 25 % of the undergraduate & PhD students group state to know what a DMP is.

 We included this additional information in the revised Figure 6a. In the main figure, we kept the cumulative representation of all 86 answers to the following questions because the absolute numbers of respondents in each career level group are in part very small (e.g., only 13 persons in the undergraduate & PhD student group). We added the per-group analysis of these questions to the supplementary information (new Suppl. Figure 9b, Extended Data 1).

**Minor issue 3:**

 Questions regarding the willingness of respondents to fulfil data management requirements (as discussed e.g. in fig. 6, 7 and 8, as well as supplemental figure 11a: In political polling, it has been demonstrated that asking voters about their intentions, rather than their expectations, leads to worse outcome predictions, likely not only because respondents are drawn towards describing own behavior with the perceived politically appropriate option, they also tend to include the prevailing opinions of their social circle when asked about expectations. For instance, see Murr, A., Stegmaier, M., & Lewis-Beck, M. (2021). Vote Expectations Versus Vote Intentions: Rival Forecasting Strategies. British Journal of Political Science, 51(1), 60-67.

 For the questions about views and behaviour regarding RDM, expectation questions could have added another perspective, and perhaps helped to mitigate the bias inherent in the recruitment. We realize, however, that this can't be added post-hoc and is intended as a remark for future studies.

**Answer:**

 We thank the reviewer for the excellent suggestion. Rephrased or additional questions in a follow-up survey might increase the reliability of the prediction of participants‘ actions instead of intentions, which might also help to mitigate bias. This aspect should be considered in future studies.
